# Surveillance of endemic human Coronaviruses in Germany, 2019/2020

**DOI:** 10.1016/j.lanepe.2021.100262

**Published:** 2021-11-04

**Authors:** Barbara Biere, Djin-Ye Oh, Thorsten Wolff, Ralf Dürrwald

**Affiliations:** Unit 17: Influenza and Other Respiratory Viruses | German National Influenza Center, Department of Infectious Diseases, Robert Koch Institute, Seestr. 10, D-13353 Berlin, Germany

The COVID-19 pandemic has renewed interest in endemic human coronaviruses (HCoV), which are responsible for an estimated 5-10% of acute respiratory infections (ARI) in temperate climate zones [1]. The epidemiological profiles of these agents may hold important clues to an anticipated future where SARS-CoV-2 has transitioned to endemicity [2], as they share physicochemical properties and route of transmission. However, due to an underappreciation of their clinical and epidemiological impact, systematic surveillance data describing the epidemiology of endemic coronaviruses NL63, HKU1, OC43 and 229E and their susceptibility to non-pharmaceutical interventions (NPI) are currently sparse. This led us to retrospectively examine our 2019/20 sample compilation, which was collected for the purpose of national surveillance of respiratory viruses in Germany as described earlier [3], providing insight into the epidemiology of endemic coronavirus infections at the German national scale for the first time.

A total of 4,375 specimens from children and adults, who had presented with acute respiratory symptoms to a sentinel physician between October 2019 and September 2020, were examined by qPCR [see supplementary information online]. Endemic coronaviruses of all four types were detected in 194 (4·4%) of the samples (Figure 1a). They were detected in all age groups from newborns to the elderly (Figure 1b). HKU1 was predominant, representing more than 55% of total coronavirus detections and peaking in December, when almost 8% of all samples contained this virus. The early HKU1 wave was followed by a less pronounced NL63 wave, indicating substantial spread with up to nearly 4% positive results in March. OC43 showed a diffuse, low level circulation between October and March, with a slight accentuation in early winter months, and 229E was detected only sporadically in winter and early spring. The non-pharmaceutical interventions (NPIs) implemented in March/April 2020 to curb SARS-CoV-2 spread [3] did appear to contribute to the decrease of endemic coronavirus detections in April, and only sporadic detections in May and June were observed throughout the remainder of the season 2019/2020. Our data indicate frequent circulation of endemic human coronaviruses from late autumn to spring, with a sharp increase in autumn and peaking from December to April, thus confirming recent reports on the temporal HCoV circulation patterns in temperate regions with a clear preponderance during the winter season [1,4,5]. Also, the decrease of virus circulation after the introduction of NPIs is compatible with the pattern observed for other respiratory viruses, although other causes cannot be excluded, as discussed earlier [3]. More insight into the natural epidemiology of these agents will be gained by their systematic surveillance at regional, national and even global levels in the future.

**Figure 1:**
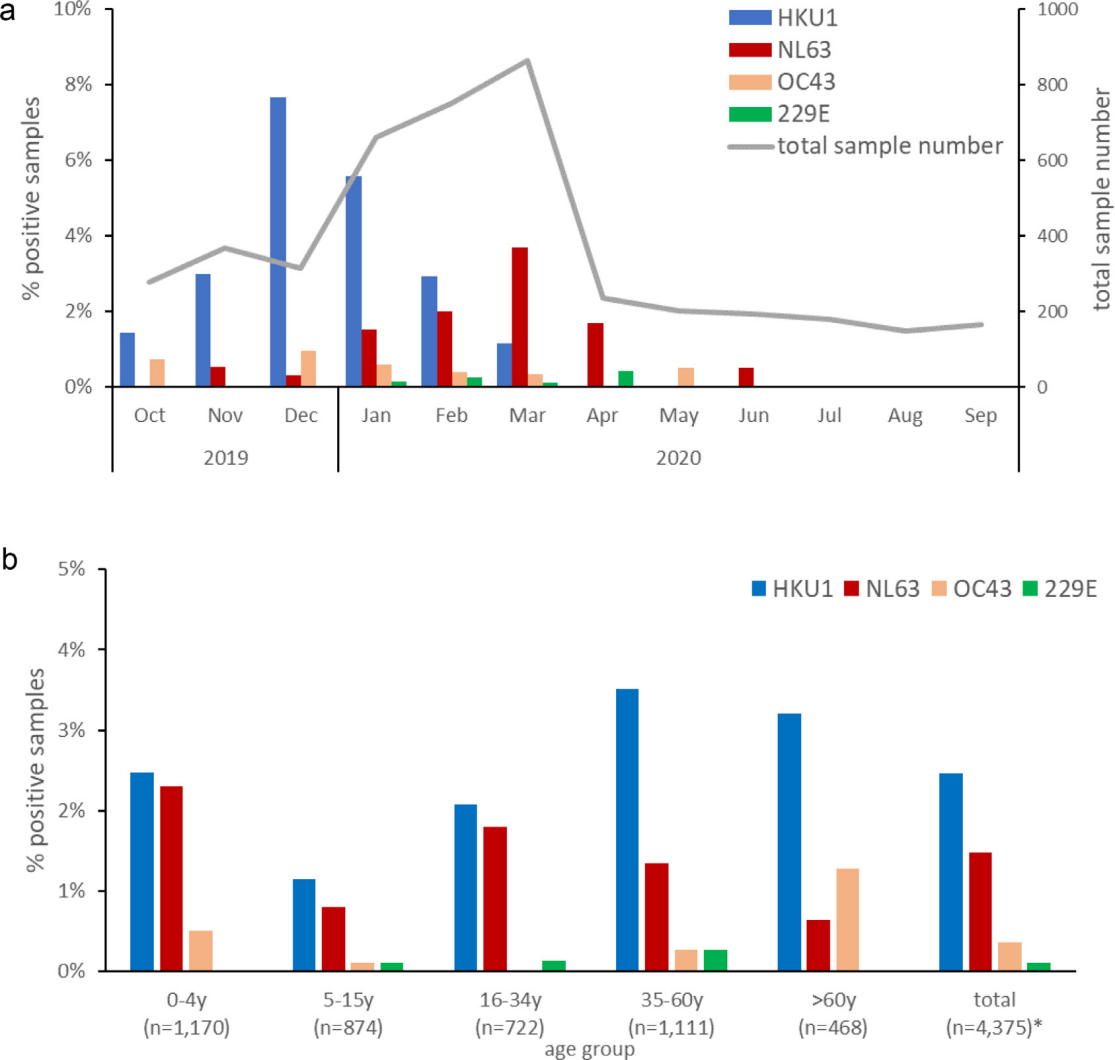
(a) virus detections for the coronavirus types per calendar month and (b) virus detections per predefined AGI age group (* including 30 samples of unknown age group)

## Contributors

BB wrote the manuscript with support from DYO and RD; BB is responsible for data acquisiton; BB and DYO analysed the data; RD is responsible for the virological section of the sentinel system; TW and RD supervised the project.

## Declaration of interests

The authors declare that no competing interests exist.

## Appendix Supplementary materials

Image, application 1

## Footnotes

Supplementary material associated with this article can be found, in the online version, at doi:10.1016/j.lanepe.2021.100262.
