# Supplementary material for: Surveillance of endemic human Coronaviruses in Germany, 2019/2020
Source: Lancet Reg Health Eur. 2021 Nov 4;11:100262. doi: 10.1016/j.lanepe.2021.100262 (PMC8566015; doi:10.1016/j.lanepe.2021.100262)
Supplement: Supplementary file 1 [file mmc1.docx]

**Supplement**

**Background on National Surveillance of Respiratory Viruses**

The surveillance of respiratory viruses in Germany is conducted by the Arbeitsgemeinschaft Influenza (AGI) and coordinated by the national public health institution, the Robert Koch Institute. Approximately 600 medical and pediatric practices participate in the network, which are distributed over the entire German territory and represent >1% of the German population. In addition to the epidemiological syndromic surveillance, a subset of practices takes nasal or pharyngeal swab samples from patients with acute respiratory infections (ARI) or influenza-like illness (ILI) for the purpose of virus identification and characterization at the German National Influenza Center at the Robert Koch Institute. Originally set up for influenza virus surveillance, the scope has been broadened to include other important respiratory viral pathogens, namely Respiratory Syncytial Virus, Human Metapneumovirus, Human Rhinovirus and Human Parainfluenzavirus (now Human Orthorubulavirus and Human Respirovirus). This system was complemented with the surveillance of SARS-CoV-2 in February 2020, shortly after its first detection in Germany.

For all samples, a written consent was given for their inclusion in research studies. All analyses were done with pseudonymised data.

**Material and Methods**

Routinely, swabs are washed out after addition of 3mL cell culture medium at the day of their arrival in the lab. RNA is extracted from 200µL sample material and subsequently undergoes cDNA synthesis. After 1:1 dilution with H_2_O, cDNA is examined by qPCR for our routine panel of respiratory viruses, including an internal control reaction,^1^ and stored at -40°C afterwards.

Retrospective coronavirus qPCR was performed on sample cDNA that had been stored for a maximum of 9 months and after an additional 1:1 sample dilution with H_2_O, following a protocol implying a triplex qPCR for NL63/229E/OC43 detection as well as a singleplex HKU1 qPCR system. Shortly, qPCR was performed in a 15µL reaction volume on LC480II real-time PCR thermal cyclers (Roche, Switzerland) in 384-well plates and applying the Platinum Taq chemistry (Thermo Fisher Scientific, USA), and 5µL of prediluted cDNA.

PCR assays were validated following the MIQE guidelines.^2^ They are routinely checked for oligonucleotide fit to current viral sequences at least once a year and adapted, if necessary.

References

1. Oh DY, Buda S, Biere B, et al. Trends in respiratory virus circulation following COVID-19-targeted nonpharmaceutical interventions in Germany, January - September 2020: Analysis of national surveillance data. *The Lancet regional health Europe* 2021; **6**: 100112.

2. Johnson G, Nour AA, Nolan T, Huggett J, Bustin S. Minimum information necessary for quantitative real-time PCR experiments. *Methods in molecular biology (Clifton, NJ)* 2014; **1160**: 5-17.
